# Accuracy of digital inclinometers for measuring knee extension during total knee arthroplasty: comparison with visual estimation and computer navigation

**DOI:** 10.1186/s42836-026-00392-9

**Published:** 2026-05-12

**Authors:** Anuwat Pongkunakorn, Chonlathan Iamsumang, Sayun Sumethvanich, Siripong Tahwang, Phatcharapon Udomluck, Rukthanin Ruktrakul

**Affiliations:** 1 Department of Orthopaedic Surgery, Lampang Hospital and Medical Education Center, Lampang, 52000 Thailand; 2 Department of Orthopaedic Surgery, Faculty of Medicine, Phayao University, Phayao, 56000 Thailand

**Keywords:** Total knee arthroplasty, Knee extension angle, Digital inclinometer, Visual estimation, Sagittal alignment, Computer navigation

## Abstract

**Background:**

Accurate intra-operative assessment of knee extension angle is essential in total knee arthroplasty (TKA), as residual flexion contracture or hyperextension is associated with inferior post-operative function and increased implant wear. Visual estimation remains widely used, but prior research has demonstrated substantial inaccuracy. This study evaluated the accuracy and reliability of a digital inclinometer technique for measuring knee extension angle during TKA, using computer navigation as the reference standard.

**Methods:**

A prospective comparative study was performed on 130 knees undergoing primary posterior-stabilized TKA. After trial implant insertion, knee extension was assessed using two methods: visual estimation and a digital inclinometer method utilizing the femoral chisel holder and anterior border of the tibial crest. All measurements were compared with OrthoPilot navigation. Accuracy was defined as measurements within ± 2° of navigation. Correlation, agreement, and intra- and inter-observer reliability were evaluated.

**Results:**

Visual estimation underestimated extension and showed a mean difference of − 3.5° ± 4.1° versus navigation (*p* < 0.001). The proportion of knees within ± 2° of navigation was only 26.2%. In comparison, the digital inclinometer demonstrated a significantly smaller mean difference of 0.1° ± 1.6° (*p* = 0.753), with 86.9% of knees meeting the ± 2° accuracy threshold. Superiority of the inclinometer persisted across all subgroups of pre-operative deformity. Correlation with navigation was weak for visual estimation (*r* = 0.320; *r*^2^ = 0.102), but very strong for the inclinometer technique (*r* = 0.894; *r*^2^ = 0.799; both *p* < 0.001). Bland–Altman analysis demonstrated minimal bias and no proportional error for the inclinometer, whereas visual estimation showed wide limits of agreement. Intra- and inter-observer reliability for inclinometer measurements was excellent (ICCs of 0.91 and 0.93), superior to visual estimation.

**Conclusion:**

The digital inclinometer technique provides accurate and reproducible intra-operative knee extension measurements, outperforming visual estimation and approximating navigation-based assessment. This low-cost approach is feasible where navigation is unavailable.

**Trial registration:**

Thai Clinical Trials Registry (TCTR20180828002). Registered on 28 August 2018. Prospectively registered.

## Introduction

Intra-operative evaluation of sagittal-plane limb alignment is a fundamental component of total knee arthroplasty (TKA). Range of motion after TKA is a key functional measure, and leaving the knee in residual flexion or hyperextension may result in suboptimal post-operative function [1]. Attainment of full knee extension, or complete correction of flexion contracture, at the conclusion of TKA is a critical determinant of post-operative performance and long-term implant survival [2]. Persistent flexion contracture produces sagittal malalignment, leading to increased quadriceps workload, elevated patellofemoral joint reaction forces, and abnormal tibiofemoral contact stresses; these biomechanical alterations are associated with inferior functional outcomes and accelerated polyethylene wear [3, 4]. In contrast, post-operative hyperextension frequently indicates inadequate soft-tissue balancing. Appropriate ligament tension and symmetric flexion–extension gaps are essential for implant durability, whereas soft-tissue imbalance remains a major contributor to early revision surgery [5]. Recent evidence demonstrates that achieving near-neutral extension (0°–5°) following TKA is associated with superior functional outcomes compared with leaving residual recurvatum [6]. Consequently, objective intra-operative verification of final knee extension angle prior to wound closure is essential to minimize implant wear and instability and to enhance long-term prosthesis survival.

Historically, intraoperative assessment of the knee extension angle or flexion contracture has relied on visual inspection. However, dependence on clinical judgment and visual estimation in the sagittal plane can lead to substantial inaccuracies. Shetty et al. [7] reported that 44% of surgeons deviated by more than 5°, and 6% deviated by more than 10° when positioning the knee at 0° extension. Similarly, Gallie et al. observed an average measurement error of 6° using this approach [8]. A standard goniometer can be used intra-operatively, but goniometer measurements significantly underestimate the knee angle at full extension compared with computer navigation, with an average difference of 2.3° (SD 4.8°); 26% of cases showed a mean difference ≥ 5°, particularly in patients with high BMI [1].

Computer navigation is considered a reliable tool for intra-operative sagittal alignment assessment during TKA, with reported accuracy within 1° [9]. Although computer-assisted navigation and robotic systems allow precise intra-operative measurement of knee extension and flexion angles, their availability remains limited in many institutions due to cost, infrastructure requirements, and increased operative time. Therefore, a simple, inexpensive, and objective intra-operative method for assessing knee extension could be valuable, particularly in conventional total knee arthroplasty without navigation. Mechanical inclinometers attached to a drill pin inserted into the trial femoral component have been used to assess knee extension intra-operatively [10]. In addition, a recent study showed that the anterior cortical line of the tibia provides an accurate reference for determining the knee extension angle relative to the sagittal mechanical axis (SMA) [11]; this line corresponds to the palpable anterior border of the tibial crest and is accessible during surgery. We adapted these concepts into a digital inclinometer method for intra-operative knee extension measurement in TKA. Digital inclinometers report accuracy of ± 0.2° [12], surpassing mechanical devices. This study assessed the accuracy of this method against conventional visual estimation.

## Methods

### Patient population

A prospective comparative study was conducted on 136 patients (136 knees) with primary knee osteoarthritis or rheumatoid arthritis who underwent primary TKA using a posterior-stabilized (PS) knee prosthesis at our institute between August 2018 and June 2021. Patients with a prior femoral fracture (1 knee) or prior tibial fracture (1 knee) were excluded. Post-operative radiographs of the remaining knees were then reviewed to determine the sagittal alignment of the femoral component. Knees demonstrating a femoral component flexion (FCF) angle outside ± 1° were excluded (4 knees, 3.0% of the initially enrolled cases). These cases were excluded to minimize the potential confounding effect of sagittal femoral component malalignment on intraoperative knee extension measurements. Excessive flexion or extension of the femoral component may alter the extension gap and therefore influence the measured knee extension angle independently of the measurement method being studied.

### Operative technique

A single experienced surgeon, who had previously performed approximately 70 navigated TKA procedures, conducted all surgeries. Following surgical exposure via the medial parapatellar approach, femoral and tibial transmitters were secured to the femur and tibia using bicortical screws and a rigid body, positioned 10–15 cm from the joint line. Anatomical landmarks and malleolar surfaces were identified using a navigation pointer and registered into the computer-assisted navigation system (OrthoPilot KneeSuite TKA 5.1, B. Braun-Aesculap, Tuttlingen, Germany). OrthoPilot intra-operative sagittal measurements have been shown to agree with post-operative lateral radiographs, with a precision of 1.4°–1.6° [13].

All PS knee prostheses used in this study were fixed-bearing designs from the Vega System® (B. Braun-Aesculap). The target extension angle for all knees was 0°–5°.

The femoral AP cutting box was set at 0° parallel to the femoral SMA, as verified by OrthoPilot. After the trial, femoral and tibial implants were placed onto the prepared bony surfaces, and a polyethylene trial was inserted between them. The femoral box chisel guide was then attached to the trial implant, and the femoral and tibial tracker arrays were secured onto their fixation arms. A bedside privacy screen was placed in front of the OrthoPilot monitor to blind the surgeons.

**Fig. 1:**
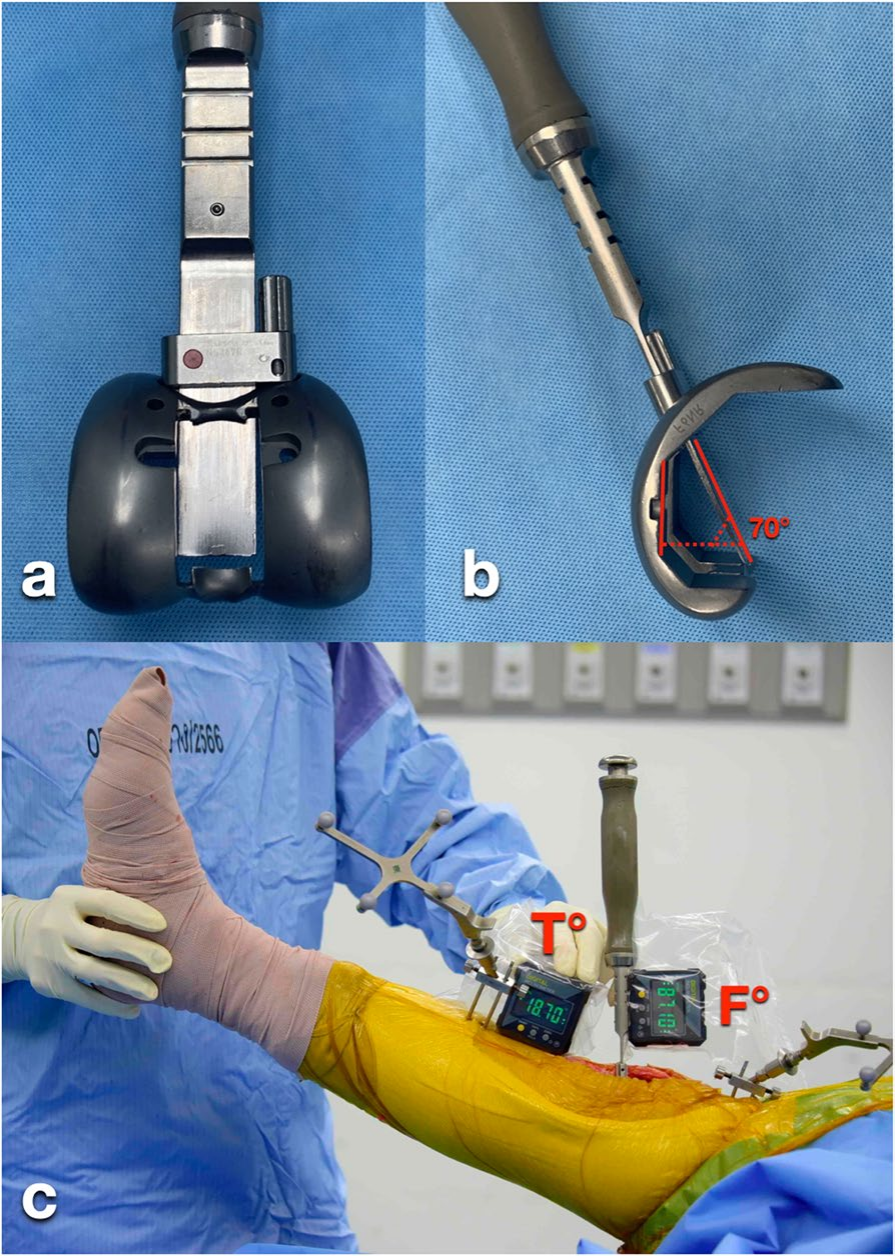
After placing the trial femoral and tibial implants with the polyethylene trial, the femoral box chisel guide was attached, and the chisel was inserted through the femoral housing slot (**a–b**). A digital inclinometer on the chisel holder measured the F angle, while another on the tibial crest measured the T angle with the leg elevated to 30°. Knee extension was calculated as (F − 70) − T°, and OrthoPilot data were recorded for comparison (**c**)

With the leg elevated to approximately 30°, gravity-assisted extension was applied. An attending surgeon (AP) and an orthopaedic resident assisting the procedure each visually estimated the angle between the thigh and leg axes and verbally reported the knee extension angle to the circulating nurse; the assessors were blinded to each other’s estimates. Immediately thereafter, the base of the first digital inclinometer was placed on the flat surface of the chisel holder to measure inclination relative to the floor, recorded as the F angle. Simultaneously, the second digital inclinometer was positioned against the anterior border of the tibial crest [14, 15], and its inclination was recorded as the T angle (Fig. 1). The knee extension angle was calculated as the difference between the femoral inclinometer reading (F) and the tibial reference angle (T), according to the geometric configuration of the prosthesis system. Specifically, the knee extension angle was calculated as (F − 70) − T degrees. The total measurement time was approximately 1 min. The inclinometer method was performed twice by the attending surgeon and twice by the resident, and all four measurements were recorded.

During all estimates and measurements, the circulating nurse recorded the hip–knee–angle alignment and the extension angle displayed on the monitor and continuously monitored this angle to cue the scrub nurse to elevate the leg, ensuring the same extension angle before each measurement. For each patient, the mean of the two visual estimates and the mean of the four inclinometer measurements were used for analysis.

### Primary and secondary study outcomes

The primary objective of this study was to assess the accuracy of the inclinometer method in measuring knee extension angles during TKA compared with the visual estimation method, using the OrthoPilot navigation system as the reference standard. Additionally, the proportion of knees exhibiting a difference greater than 2° between the two methods was calculated, with this threshold considered clinically significant based on the reported ± 1° accuracy of the navigation system [16]. Angle overestimation and underestimation were defined when the estimated extension angle was above and below the OrthoPilot measurement by more than 1°, respectively. The primary outcome was the proportion of knee extension angle measurements with an error ≤ 2°. The secondary outcome was the degree of deviation from the reference angles reported by OrthoPilot.

At 1 year post-operatively, a true lateral cross-table knee radiograph and a standing long-leg hip-to-ankle anteroposterior radiograph were obtained using the same protocol as pre-operatively. The angle between the anterior cortical line of the tibia (ACLT) and the anterior cortical line of the femur (ACLF) was measured at both time points to document initial and residual flexion contracture or hyperextension [11]. The hip–knee–ankle angle (HKAA) was measured to assess coronal mechanical alignment pre- and post-operatively. On a short lateral knee radiograph, the ACLF was drawn along the distal anterior cortex using points 5 and 10 cm above the joint line to approximate the femoral sagittal mechanical axis defined by OrthoPilot (mean difference 0.0°, SD 1.9°) [17]. The femoral component sagittal axis (FCSA) was the perpendicular line to the distal rear surface of the distal femoral component [18]. The angle between the ACLF and FCSA was the femoral component flexion angle (FCFA); positive values indicate flexion and negative values indicate extension.

Two orthopaedic surgeons, blinded to each other’s results, performed all radiographic measurements. The assessments were repeated two weeks later, and the mean of the four readings was used for analysis.

### Data analysis

The Shapiro–Wilk test was applied to assess data normality before further analysis. Angle measurements from both methods, as well as each method compared with OrthoPilot, were analyzed using paired t-tests. Categorical data were analyzed with the exact probability test. The correlations between knee extension angles measured by the inclinometer and OrthoPilot navigation system, and between the visual estimation method and OrthoPilot, were assessed using the Pearson correlation coefficient. Bland–Altman plots were used to illustrate the differences in angle measurement between the inclinometer method and OrthoPilot. The intraclass correlation coefficients (ICC) with the 2-way random- effects model and absolute agreement were calculated for intra- observer and interobserver reliability. All statistical analyses were conducted using STATA software, version 12.1 (StataCorp LP, USA) and SPSS version 27 (SPSS Inc, Chicago, USA). A *p*-value of less than 0.05 was considered statistically significant.

The sample size was determined to detect a significant difference in the proportion of knees with an intra-operative estimation error ≤ 2°. In our pilot study of 25 knees assessed using the visual estimation technique, 32% (8 knees) were within this threshold compared with Orthopilot. We hypothesized that the inclinometer method would achieve accuracy within 2° in 70% of knees. Using a two-sided type I error rate of 0.01 and 95% statistical power, the required sample size calculated with the two-dependent proportions formula was 119 knees [19]. The study obtained ethical approval (Code 93/61) and was registered (ID: TCTR20180828002).

This study was approved by the institutional review board and registered with the Thai Clinical Trials Registry (TCTR20180828002). Written informed consent was obtained from all participants prior to enrollment.

## Results

Among 130 patients (130 knees), 78.5% were women (102/130). Mean age was 62.5 ± 4.3 years (range, 51–71), and mean BMI was 25.0 ± 3.9 kg/m^2^ (range, 16.4–36.1). Primary osteoarthritis accounted for 98.5% of cases (128/130). Most knees were Kellgren–Lawrence grade 4 (93.1%). Pre-operative hip–knee–ankle alignment demonstrated varus deformity with a median of 7° varus (IQR, 3°–9° varus; range, 20° varus to 17° valgus), which improved to 1° varus (interquartile range [IQR], 0°–3° varus) post-operatively. Pre-operative knee extension angles were 5.7° ± 6.8° (range, − 8° to 25°), which improved post-operatively to 1.6° ± 3.7° (range, − 6° to 9°). All femoral components were implanted nearly parallel to the SMA, with a median FCFA of 0.25° [IQR 0.0°, 0.75°] (range, − 1° to 1°) (Table 1).

**Table 1:** Baseline characteristics and radiographic data of the patients

Characteristic	Value (*n* = 130)
**Gender, (women/men), *n* (%)**	102 (78.5)/28 (21.5)
**Age (yr), mean ± SD**	62.5 ± 4.3
Range	51–71
**BMI (kg/**^**2**^**), mean ± SD**	25.0 ± 3.9
Range	16.4–36.1
**Diagnosis, *n* (%)**	
Primary osteoarthritis	128 (98.5)
Rheumatoid arthritis	2 (1.5)
**Kellgren and Lawrence grading, *n* (%)**	
Grade 4	121 (93.1)
Grade 3	9 (6.9)
**Hip–knee–ankle angle**	
Pre-operative median (IQR)	7° varus (3° to 9° varus)
Range	20° varus to 17° valgus
Post-operative median (IQR)	1° varus (3° to 0° varus)
Range	4° varus to 4° valgus
**Extension angle**	
Pre-operative mean ± SD	5.7° ± 6.8°
Range	− 8° to 25°
Post-operative mean ± SD	1.6° ± 3.7°
Range	− 6° to 9°
**Femoral component flexion angle median [IQR]**	0.25° [0.0°, 0.75°]
Range	− 1° to 1°

After trial implant insertion, the mean knee extension angle was 0.1° ± 3.5° (range, − 10° to 10°) by visual estimation, 3.7° ± 3.6° (range, − 5° to 14°) by the inclinometer method, and 3.6° ± 3.5° (range, − 4° to 11°) by OrthoPilot. There was no significant difference between the inclinometer method and OrthoPilot (*p* = 0.753). When stratified by pre-operative extension angle (< 0°, 0°–10°, and > 10°), the inclinometer measurements showed no statistically significant difference to the OrthoPilot values across all subgroups (*p* > 0.05, for all) (Table 2). However, visual estimation yielded significantly lower values compared with both the inclinometer method (*p* < 0.001) and OrthoPiloAt (*p* < 0.001).

**Table 2:** Knee extension angles after trial implant insertion, compared across three measurement methods and stratified by pre-operative extension category determined from cross-table lateral radiographs (*n* = 130)

Pre-operative extension angle	Measurement methods			*p*-value		
	**Visual**	**Inclinometer**	**OrthoPilot**	**Visual vs. Inclinometer**	**Visual vs. OrthoPilot**	**Inclinometer vs. OrthoPilot**
**All cases (*n* = 130)**						
Mean ± SD	0.1° ± 3.5°	3.7° ± 3.6°	3.6° ± 3.5°	** < 0.001**	** < 0.001**	0.753
Range	− 10° to 10°	− 5° to 14°	− 4° to 11°			
** < 0° (*n* = 13)**						
Mean ± SD	− 1.2° ± 3.6°	2.2° ± 4.2°	2.3° ± 4.0°	** < 0.001**	** < 0.001**	0.955
**0°–10° (*n* = 59)**						
Mean ± SD	0.0° ± 3.5°	3.2° ± 3.2°	3.0° ± 3.2°	** < 0.001**	** < 0.001**	0.761
** > 10° (*n* = 58)**						
Mean ± SD	0.4° ± 3.5°	4.7° ± 3.7°	4.5° ± 3.6°	** < 0.001**	** < 0.001**	0.699

**Fig. 2:**
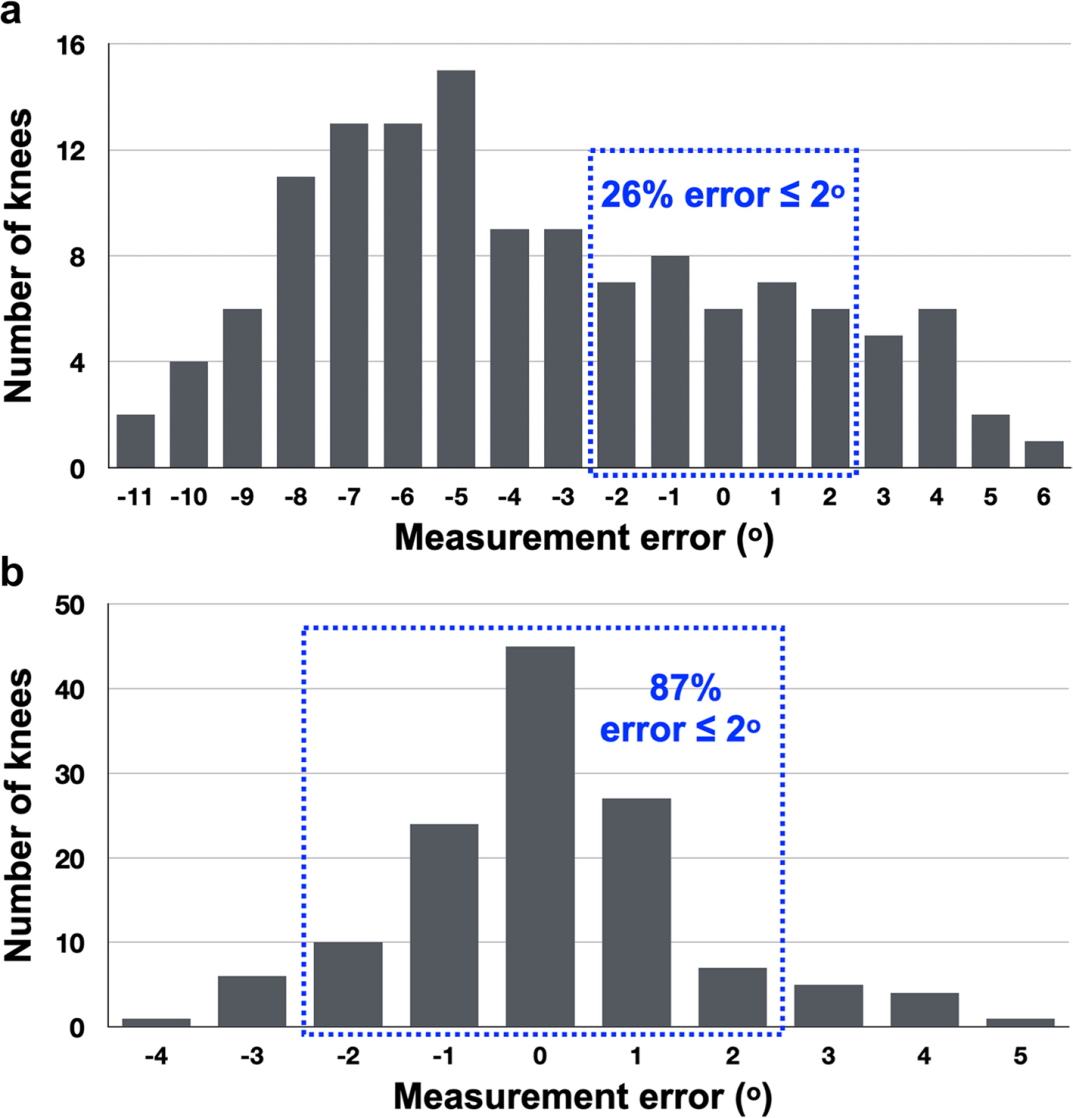
Distributions of knee extension angle differences between visual estimation and OrthoPilot (**a**), and between the inclinometer and OrthoPilot (**b**). The dotted line marks the ± 2° threshold; the percentage of knees within this range is reported

The mean difference between the visual estimation method and OrthoPilot was − 3.5° ± 4.1° (range, − 11° to 6°; 95% CI, − 4.2° to − 2.8°), with 26.2% of knees (34/130) falling within 2° of OrthoPilot (34/130 knees) (Fig. 2a). In contrast, the mean difference between the inclinometer method and OrthoPilot was 0.1° ± 1.6° (range, − 4° to 5°; 95% CI, − 0.2° to 0.4°), with 86.9% of knees (113/130) showing discrepancies within 2° (Tables 2 and 3; Fig. 2b). This superiority in accuracy was maintained across all pre-operative deformity subgroups.

**Table 3:** Accuracy of two measurement methods after trial implant insertion, reported as the proportion of knees within 2° of OrthoPilot, stratified by pre-operative extension category determined from cross-table lateral radiographs (*n* = 130)

Pre-operative extension angle	Accuracy		*p*-value
	**Visual estimation, *n* (%)**	**Inclinometer, *n* (%)**	
**All cases** (*n* = 130)	34 (26.2)	113 (86.9)	** < 0.001**
** < 0°** (*n* = 13)	3 (23.1)	11 (84.6)	** < 0.001**
**0°–10°** (*n* = 59)	16 (27.1)	52 (88.1)	** < 0.001**
** > 10°** (*n* = 58)	14 (25.9)	50 (86.2)	** < 0.001**

**Fig. 3:**
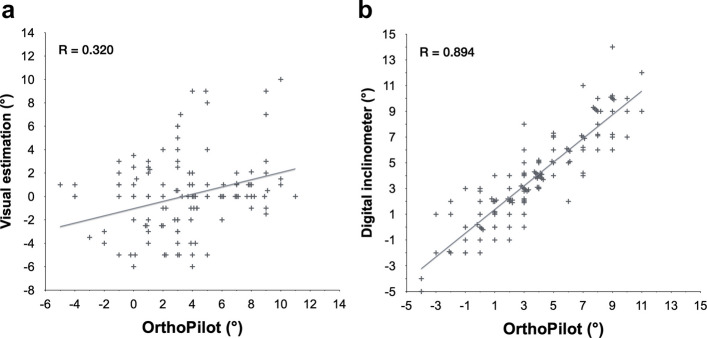
(**a**) Weak positive correlation between visual estimation and OrthoPilot (*r* = 0.320); (**b**) Very strong positive correlation between the digital inclinometer and OrthoPilot (*r* = 0.894). In panel (b), the solid line shows the least-squares regression; *r*^2^ = 0.799, indicating that approximately 80% of the variance in inclinometer readings is explained by OrthoPilot. Data points lie close to the fitted line with relatively uniform scatter

Correlation analysis revealed a weak positive correlation was found between visual estimation and OrthoPilot measurements, with a Pearson correlation coefficient of *r* = 0.320 (95% CI 0.167 − 0.441, *p* < 0.001) (Fig. 3a).* T*he inclinometer method demonstrated a very strong positive correlation with OrthoPilot (*r* = 0.894, 95% CI 0.854 − 0.925, *p* < 0.001), with the regression model explaining approximately 80% of the variance (*r*^2^ = 0.799) (Fig. 3b).

**Fig. 4:**
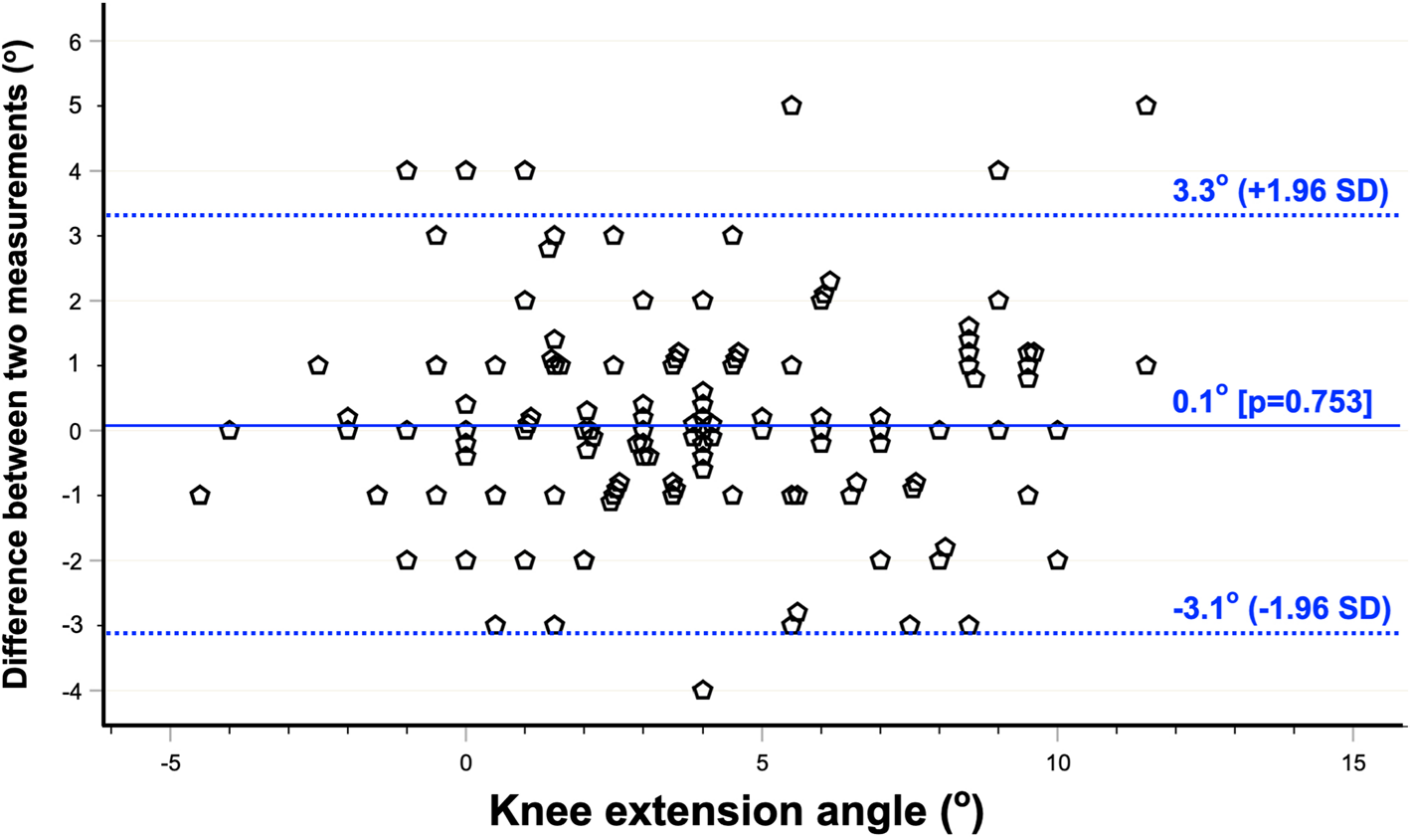
Bland–Altman plot for agreement between digital inclinometer and OrthoPilotknee extension angles. The solid blue line is the mean difference (bias) of 0.1° (*p* = 0.753), indicating no systematic offset. The dotted blue lines are the 95% limits of agreement

The Bland–Altman analysis of the inclinometer method showed a mean difference (bias) of 0.1° relative to OrthoPilot (*p* = 0.753), indicating no systematic offset. The data points were evenly distributed around the bias line across the full measurement range, with no discernible upward or downward trend, suggesting no proportional bias and further supporting the reliability of this measurement method (Fig. 4).

Reliability analysis demonstrated good to excellent agreement for all parameters, with ICC values ranging from 0.76 to 0.97. Intra-operatively, the digital inclinometer yielded superior reliability (intra-observer ICC = 0.91, inter-observer ICC = 0.93) compared to visual estimation (inter-observer ICC = 0.76) (Table 4).

**Table 4:** ICC values for intra-observer and inter-observer reliability of measurement results

Measurement	Intra-observer ICC	Inter-observer ICC
**Intra-operative extension angle**		
Visual estimation	NA*	0.76
Inclinometer	0.91	0.93
**Radiographic measurement**		
Hip–knee–ankle angle	0.91	0.87
Pre- and post-operative extension angle	0.84	0.86
Femoral component flexion angle	0.92	0.97

^*^NA: Not Applicable

A minimum follow-up period of two years was completed for all patients. No peri-prosthetic joint infections were reported during this period.

## Discussion

This study demonstrated that measuring knee extension angles using a digital inclinometer placed on the chisel holder connected to the trial femoral component, in combination with the anterior border of the tibial crest, provides high accuracy comparable to the OrthoPilot navigation system during TKA. The mean difference between the inclinometer and navigation measurements was only 0.1°, indicating excellent agreement. Furthermore, the inclinometer method achieved clinically acceptable accuracy (error ≤ 2° of navigation) in 86.9% of cases. The high Pearson correlation coefficient and the narrow limits of agreement in the Bland–Altman analysis further support the reliability of this method. This accuracy is likely explained by the fixed geometric relationship between the chisel holder and the sagittal axis of the femoral component, which is parallel to the OrthoPilot femoral SMA. Similarly, the palpable anterior border of the tibial crest can represent the ACLT [14], which is nearly parallel to the OrthoPilot tibial SMA, with a mean anterior inclination of 0.3° [20–22].

This novel technique relies on precise femoral component positioning, as excessive flexion or extension can result in over- or under-estimation of the knee extension angle. In this study, the median femoral FCFA was 0.25°, indicating that the component was aligned parallel to the femoral SMA, which likely favored the performance of the OrthoPilot navigation system. In conventional TKA using an intramedullary rod for distal femoral cutting, surgeons must avoid placing the cut in excessive flexion or extension relative to the femoral SMA, as sagittal placement of the femoral component remains one of the most error-prone steps, particularly in patients with greater height, weight, or BMI [23]. Utilizing an intramedullary rod 9 mm in diameter and 9 inches in length [23], along with selecting an entry point 10 mm anterior to the intercondylar notch apex in Caucasian patients [24], or 12–15 mm in Asian patients [25, 26], can help reduce this risk.

The intra-operative use of inclinometers to assess knee extension during TKA has been reported by several investigators. Jacobs et al. [10] used two inclinometers, each equipped with a freely moving angle indicator arm and an inferior counterweight. The femoral inclinometer was attached to a drill pin inserted into the trial femoral component, while the tibial inclinometer was mounted on the handle of the trial tibial inserter. With the leg positioned in 30° of hip flexion, the knee extension angle was quantified as the angular difference between the readings of the two inclinometers. This value represented a combination of the angular difference between the femur and tibia, as well as the posterior slope of the proximal tibial cut. The device demonstrated high intra-operative reliability, detecting a 2.7° loss of knee extension following the insertion of a 2 mm-thicker trial polyethylene insert. However, the study did not include a comparison of this technique’s accuracy relative to the SMA or with computer navigation measurements. In contrast, our method utilizes digital inclinometers that rely on electronic sensors or gyroscopes to measure angles relative to gravity. Compared to mechanical inclinometers, digital devices generally provide greater resolution and precision, and their readings are faster and easier to interpret, without being affected by friction, wear, or user reading errors. The femoral inclinometer in our method was directly attached to the trial femoral component via the chisel holder, which maintains a fixed angulation relative to the sagittal axis of the femoral prosthesis, rather than to a pin inserted into a drilled hole in the component, which is intended to be perpendicular to that axis.

For the tibial measurement, the inclinometer was placed on the anterior surface of the proximal tibial crest, just distal to the skin incision. A previous study demonstrated that the anterior cortical line (ACL) of the distal femur and proximal tibia can accurately determine the knee extension angle relative to the sagittal mechanical axis during total knee arthroplasty, with a mean difference of only 0.2° compared with computer navigation measurements [11]. The ACL method relies on readily identifiable anatomical landmarks such as the anterior cortex of the distal femur and the anterior tibial crest. The inclinometer technique used in the present study follows a similar principle by referencing intra-operative anatomical landmarks through a fixed reference pin and digital inclinometer measurement. Therefore, our method may be considered a practical intra-operative application of this anatomical alignment concept without requiring fluoroscopy or navigation.

This study showed that conventional visual estimation consistently underestimated residual flexion, resulting in substantial inaccuracy, with only 26.2% of assessments falling within the ± 2° error margin. While visual estimation suggested a mean extension angle of neutral (0°), the actual angle verified by navigation was 3.5° of flexion. These findings confirm the inferiority of subjective assessment when compared with objective measurement and highlight the critical limitations of visual judgment in detecting clinically meaningful sagittal plane malalignment. This is consistent with prior evidence demonstrating that visual assessment commonly underestimates fixed flexion contracture, with a reported median error of − 4° [8, 10], likely due to challenges in reliably identifying the hip center intra-operatively, soft-tissue bulk from the extensor mechanism, and the obscuring effect of surgical drapes [1, 7].

The clinical relevance of this technique lies in its ability to objectively verify final knee extension after prosthetic implantation, as residual sagittal imbalance following TKA adversely affects clinical outcomes. At five years post-operatively, knees exhibiting hyperextension at wound closure (− 10° to 0°) or flexion contracture greater than 10° demonstrate significantly lower Knee Society Knee Scores, while hyperextension at wound closure is also associated with inferior Knee Society Functional Scores compared with the ideal extension group (0°–5°) [6]. These findings delineate a narrow therapeutic window and emphasize the importance of accurately measuring the final intra-operative extension angle at wound closure to prevent deviation into higher-risk categories. Furthermore, either a gravity-assisted extension angle between − 10° and 0° or a passive extension angle greater than 0°, measured immediately after wound closure, closely corresponds to the final knee extension maintained over follow-up periods of up to five years [6].

**Fig. 5:**
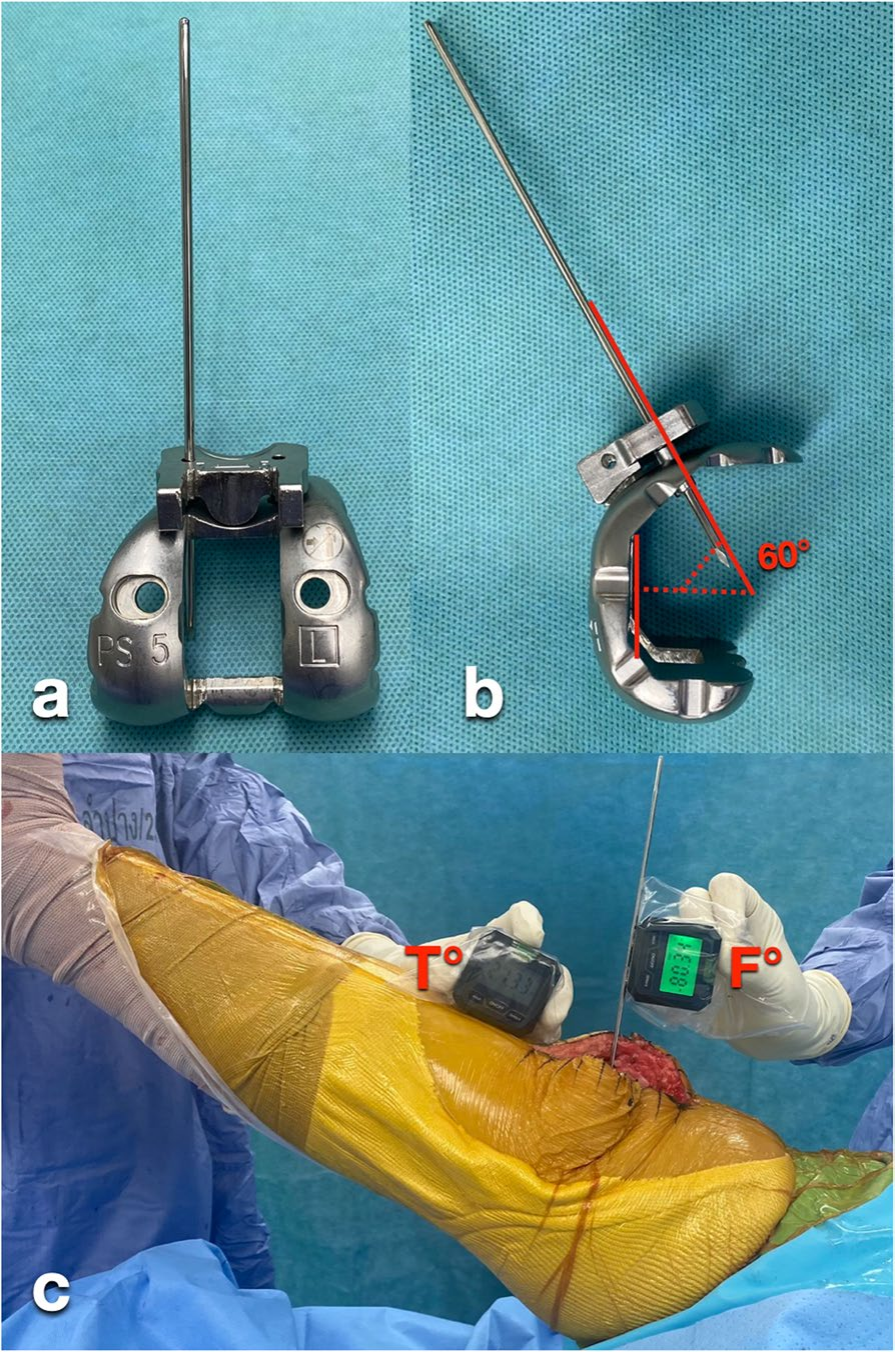
To apply the inclinometer method to the Persona Knee prosthesis system (Zimmer Biomet), the PS box cut guide was mounted onto the anterior holes of the PS femoral provisional, and a 3.2 mm Steinmann pin was inserted through one of the guide holes (**a–b**). The base of the first inclinometer was placed on the pin to measure the F angle (**c**). The knee extension angle was calculated as (F − 60) − T degrees

Our technique may be extended to other PS knee prosthesis systems that incorporate a pin or flat metal reference fixed at a known angle to the prosthetic sagittal axis. For example, in the Persona Knee prosthesis system (Zimmer Biomet, USA), the PS box cut guide can be mounted onto the anterior holes of the PS femoral provisional, allowing for the insertion of a 3.2 mm Steinmann pin through one of the guide holes. This configuration establishes a consistent angle of 60° between the pin and the sagittal axis of the femoral provisional (Fig. 5), providing a reliable reference for alignment. Such adaptability highlights the broader clinical relevance of the technique and its potential for integration into various surgical workflows.

**Fig. 6:**
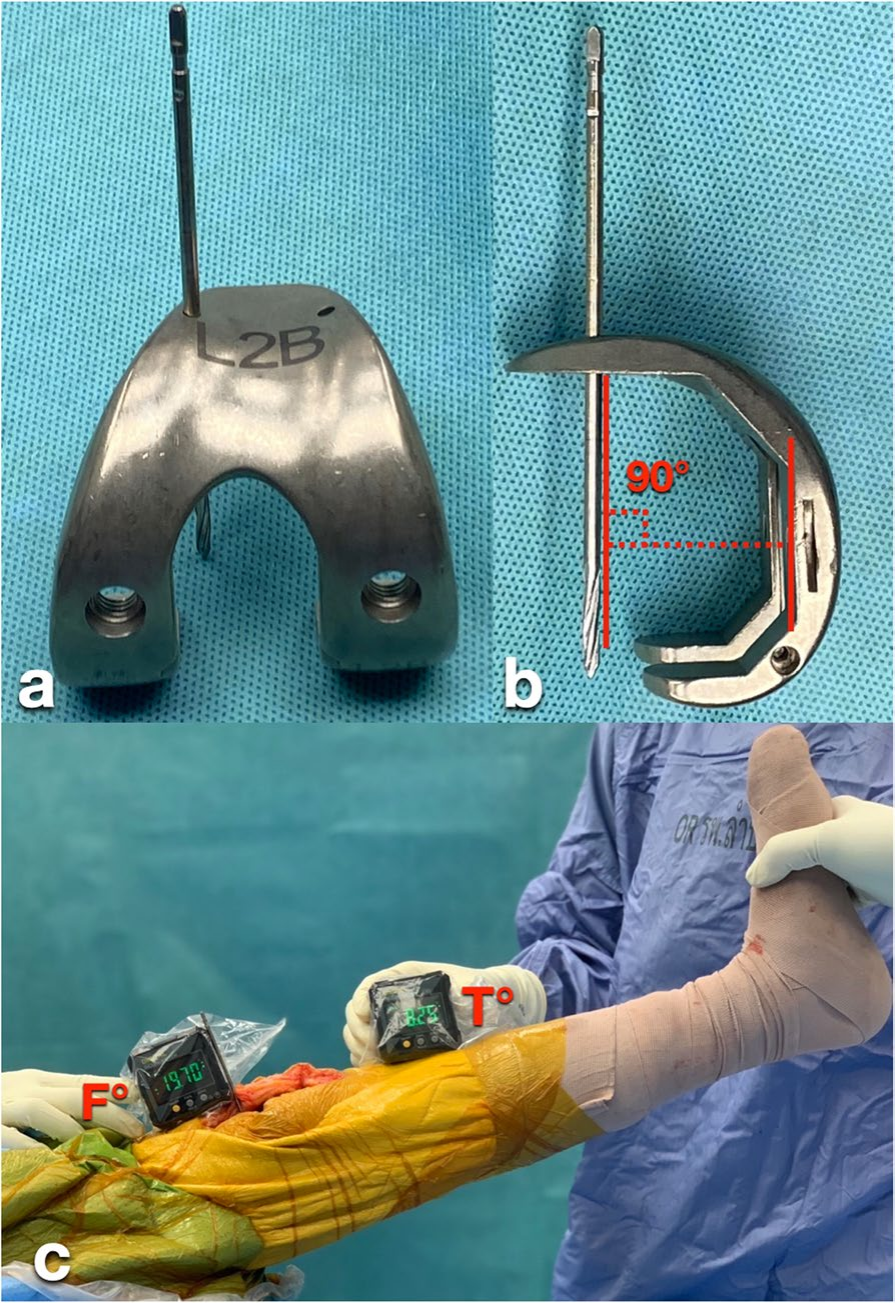
To apply the inclinometer method to the Gemini SL knee system (Waldemar Link GmbH & Co), a snugly fitted pin was inserted into a guide hole located on the anterior flange of the trial femoral component (**a–b**). The side of the first inclinometer was placed on the pin to measure the F angle (**c**). The knee extension angle was calculated as F − T degrees

Our method may also be applicable in cases where a box cut guide is not available on the trial femoral component but where a guide hole is present in the anterior flange, as seen in the Gemini SL knee system (Waldemar Link GmbH & Co, Germany). This hole is generally positioned perpendicular to the prosthetic sagittal axis, allowing a snugly fitted pin to be inserted and used as a consistent reference point. This approach aligns with the technique described by Jacobs et al. [10] (Fig. 6).

**Fig. 7:**
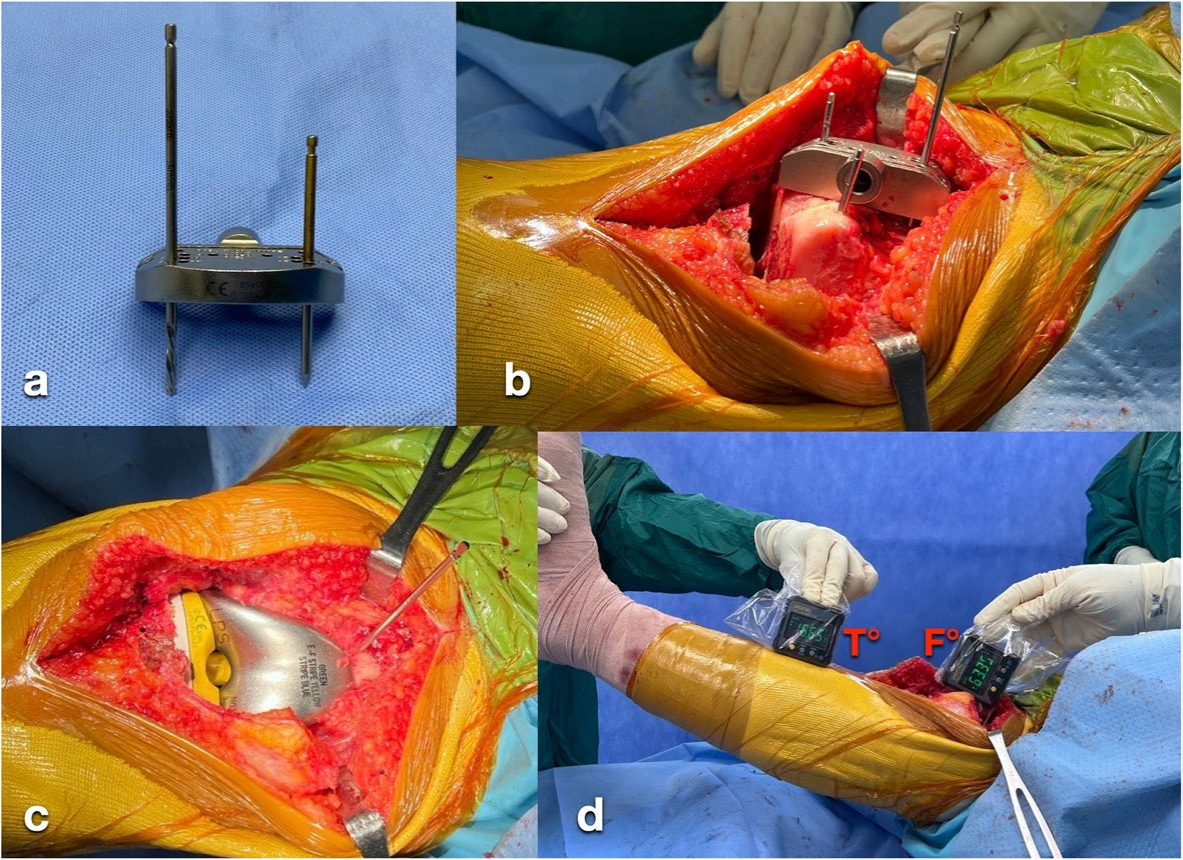
When the trial femoral component lacks a box-cut guide or anterior flange guide hole, a supracondylar reference pin is placed. After distal femoral resection, the cutting guide is released from one headless pin, rotated approximately 45° proximally, and a 3.2-mm drill bit is inserted through the “0” pin hole as the reference pin (**a–b**). The base of the first inclinometer is placed on the pin to measure the F angle (**c–d**). The knee extension angle is calculated as (F − 90) − T degrees

In situations where the trial femoral component lacks both a box-cut guide and an anterior flange guide hole—such as with cruciate-retaining designs or the NexGen LPS Knee System (Zimmer Biomet, USA)—a reference pin can be placed at the supracondylar region. After completion of the distal femoral cut, the distal femoral cutting guide is disengaged from one headless holding pin while remaining secured to the other. The guide is then rotated proximally by approximately 45°, and a 3.2-mm drill bit is inserted through the standard pin hole marked “0” on the anterior surface of the cutting guide to function as a reference pin. Attention must be paid to positioning the drill bit proximal to the anticipated most superior margin of the anterior flange to avoid prosthetic abutment after implantation. In addition, the drill bit should engage the far cortex to ensure stability and prevent intraoperative migration. The resulting reference pin is oriented perpendicular to the prosthetic sagittal axis, consistent with the previously described technique [10] (Fig. 7).

This study has certain limitations. First, the use of navigation as a reference standard has limitations. Although it provides real-time measurements, accuracy depends on proper registration and stable sensor positioning. Registration error or tracker misalignment can introduce systematic bias and may affect the validity of measurements. Second, the sagittal mechanical axis (SMA) was defined using the OrthoPilot navigation system, which identifies it as the line connecting the femoral head center to a point located 65% posteriorly between the anterior cortex and the most prominent point of the posterior medial femoral condyle. Other navigation systems may define SMA differently. Third, we did not assess sagittal femoral bowing, as full-length lateral radiographs of the femur and tibia were not obtained. Although patients with prior femoral or tibial fractures were excluded using hip-to-ankle orthoroentgenograms, unrecognized bowing may have influenced the results. Excessive sagittal bowing may lead to underestimation of knee extension angles with the inclinometer, while minimal bowing may cause overestimation [17]. This variability should be considered when interpreting the findings. Fourth, the knee prosthesis used in this study was limited to the Vega knee system, which features a slot for a chisel holder connected to the trial femoral component. Extrapolation to other knee prostheses—such as those with a PS box cut guide or an anterior flange guide hole—requires further investigation to validate the accuracy of the method. Fifth, the surgeries were performed by a single experienced surgeon at a single institution, which may limit generalizability, although the high inter-observer reliability suggests the method is readily learnable. Sixth, while we verified outcomes with navigation, we did not report long-term functional outcomes in this specific report, which warrants further investigation. Finally, the present technique measures knee extension angle rather than the complete range of knee motion. Future studies may investigate whether this method can reliably quantify both intra-operative extension and flexion angles.

## Conclusion

Intra-operative visual estimation of knee extension after trial implantation demonstrated limited accuracy when compared with navigation measurements. The digital inclinometer technique provided measurements that closely matched the navigation system while requiring minimal additional equipment. This simple and inexpensive method may serve as a useful adjunct for objectively verifying knee extension during conventional posterior-stabilized total knee arthroplasty when computer navigation or robotic systems are not available.

## Data Availability

The datasets used and analyzed in the study are available on request to the corresponding author.

## Abbreviations

ACLF: Anterior cortical line of the femur
ACLT: Anterior cortical line of the tibia
BMI: Body mass index
CI: Confidence interval
FCFA: Femoral component flexion angle
FCSA: Femoral component sagittal axis
HKAA: Hip–knee–ankle angle
ICC: Intraclass correlation coefficient
IQR: Interquartile range
PS: Posterior-stabilized
SD: Standard deviation
SMA: Sagittal mechanical axis
TKA: Total knee arthroplasty
